# Non-coronary chest pain does not affect long-term mortality: a prospective, observational study using a matched population control

**DOI:** 10.1186/s12875-016-0559-z

**Published:** 2016-11-16

**Authors:** Staffan Nilsson, Petter Järemo

**Affiliations:** 1 Primary Health Care and Department of Medicine and Health Sciences, Linköping University, Norrköping, Sweden; 2 Department of Internal Medicine, The Vrinnevi Hospital, Norrköping, Sweden; 3 Vikbolandet Health Care Centre, Bygdevägen 13, Vikbolandet, SE-610 24 Sweden

**Keywords:** Coronary heart disease, Hypertension, Mortality, Non-coronary chest pain, Primary health care

## Abstract

**Background:**

Chest pain assumed to be of non-coronary origin (NCCP) may be linked to enhanced mortality due to coronary heart disease (CHD). The aim of this study was to follow NCCP patients, as defined in primary care, with respect to mortality and long-term morbidity of CHD. We further examined if NCCP associates with risk factors for CHD.

**Methods:**

Patients consulting general practitioners (GPs) in 1998–2000 in three primary care centers in the southeast Sweden for chest pain regarded as NCCP were compared with controls matched for age, gender and residential area. Causes of death were gathered from registry data and death certificates. In 2005 a postal questionnaire was distributed to the survivors to collect demographic and clinical data. If participants had CHD diagnosed by a physician prior to inclusion they were excluded.

**Results:**

Patients with NCCP (*n* = 382) and population controls (*n* = 746) did not differ with respect to mortality and incidence of CHD. The NCCP group reported more ongoing chest pain (OR 3.34 95 % CI 2.41–4.62), they more often had elevated blood pressure (OR 1.86 95 % CI 1.32–2.60), consumed more β-blockers (*p* < 0.001), aspirin (*p* = 0.013), thiazides (*p* = 0.004) and long-acting nitrates (*p* = 0.002). They further had more remedies for acid-related disorders (*p* = 0.014) and obstructive pulmonary disease (*p* < 0.001).

**Conclusions:**

The study suggests that individuals with chest pain judged by GPs to be NCCP do not develop CHD more frequently than population controls. It is evident that NCCP often lasts for many years and that the condition associates with hypertension.

## Background

Chest pain from causes other than coronary heart disease (CHD) is widespread [1–3]. Non-specific or non-cardiac chest pain is a heterogeneous condition defined as chest pain of other cause than cardiac and not explained by other well-established conditions [2, 4]. In this article we have chosen to use the term non-coronary chest pain (NCCP) since coronary heart disease (CHD) is the most prevalent serious cause of chest pain and the number one cause that clinical evaluation and investigations aim to rule out. In primary care populations NCCP without established CHD may imply an increased risk of subsequent CHD and death [5, 6]. There are various reports on the time extension of NCCP [7, 8]. In the primary care setting a majority of patients report chest pain for more than 6 months [4]. Although NCCP-patients in some studies have a good outcome concerning mortality they may suffer a considerable morbidity [9, 10] and also impaired quality of life [2, 11, 12]. Knowledge of long term outcome of NCCP is limited and needs clarification but female NCCP sufferers have a higher prevalence of cardiovascular risk factors [13]. In a previous study [14], we defined patients consulting for chest pain that the general practitioner (GP) regarded as less likely to be due to CHD. They were followed for almost 6 years with the aim to evaluate if they develop CHD more frequently and if they have more risk factors for CHD.

## Methods

### Setting and participants

NCCP patients were selected from 1 suburban and 2 rural primary health care centres [14] serving the nearby population. The catchment areas of the health care centres were similar to the parishes as defined in the Swedish National Population Registry. Patients were included between May 1998 and April 2000 if they consulted their GP for chest pain that started in the previous 6 months and if they were between 20 and 79 years old. The inclusion involved 441 NCCP individuals and 50 patients judged as possible CHD. After further clinical investigation 21 of the latter were assessed as NCCP and thus 462 patients remained. Five of them were excluded due to a previous history of CHD. Sixty-two individuals below 35 years of age were kept out as the risk for CHD in their age group was considered to be very low. Two NCCP patients were excluded since they had protected addresses and were impossible to contact by regular mail. Due to an administrative failure one further NCCP patient was excluded. Hence, 392 NCCP patients remained for evaluation. In 2005, for each patient, two controls matched for age, gender and residency area were chosen from the Swedish National Population Registry. The database contains data of all residents in Sweden, identified by their unique personal identity number which includes date of birth. In addition, for each individual the database contains information about postal address, parish of residency and if applicable, date of decease. For each NCCP patient we chose two controls of same gender being as close as possible in age and living in the same primary health care catchment area. We did not include unmatched cases in the study. Consequently, most control pairs had similar age as their NCCP counterpart but it could differ up to 4 years. The controls were selected in 2005 and given the same inclusion date (between May 1998 and April 2000) as the corresponding case. All subjects were alive when they entered the study.

### Postal questionnaire

A postal questionnaire was used, on average 5 years and 11 months (SD ± 7 months) after the initial consultation, to collect demographic data such as education, family status and smoking habits. We inquired about chest pain within the last 6 months. Questions were also asked about acute CHD (myocardial infarctions and unstable angina pectoris), angina pectoris, increased cholesterol level and hypertension. The survey also assessed current use of medication of all kinds and consultations for chest pain in recent years.

### Validation of clinical data

Causes of death were gathered from the Cause of Death Registry and from death certificates for all deceased participants. The medical records of the deceased were checked for pre-mortem CHD. If participants reported acute CHD and/or stable angina pectoris, their medical records were reviewed. All charts were assessed blinded to group allocation. Angina pectoris was diagnosed clinically or the diagnosis was based on findings at coronary angiography, exercise testing and/or myocardial perfusion scintigraphy. Other medical conditions were not verified by inspecting medical records.

### Mortality analysis

**Fig. 1:**
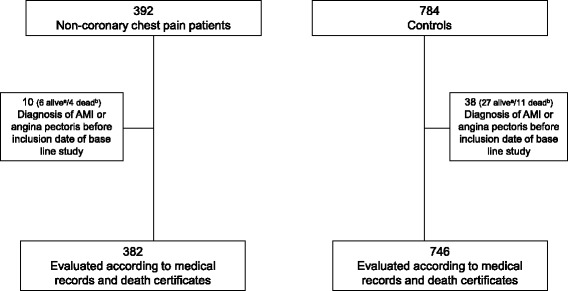
Flowchart showing the management of the 392 non-coronary chest pain (NCCP) patients and the 784 controls

The study originally included 392 patients with NCCP and 784 controls. Ten patients with NCCP (six alive and four dead) and 38 controls (27 alive and 11 dead) were excluded because medical records revealed the presence of CHD before inclusion. The remaining 382 NCCP patients and 746 controls were assessed with respect to mortality (Fig. 1).

### Basis of NCCP classification

In 1998–2000, the 382 patients with NCCP were classified as such after clinical evaluation in 208 cases, after a normal exercise test in 123 cases and after a normal myocardial perfusion study in 19 cases. In addition, 32 cases were assessed as having a low likelihood for CHD after examination at a nearby hospital.

### Managing of patients according to the postal questionnaire

A flowchart showing the management of the 392 patients with NCCP and the 784 controls is presented in Fig. 1. The survey was not sent to the families of the deceased individuals (20 NCCP patients and 39 controls). The questionnaire was delivered by regular mail to the remaining 372 patients and 745 controls. It was returned by 285 patients (77 %) and 557 controls (75 %). Of these, six individuals with assumed NCCP and 27 controls were excluded as they had CHD diagnosed before inclusion. Thus, 279 patients with NCCP and 530 controls remained to be assessed.

### Statistics

The Pearson chi-squared test and the Fisher exact test were employed for nominal variables. The unpaired two-tailed Student *t*-test was used for continuous data. Univariate logistic regression was performed for “consultations for chest pain in recent years” and clinical characteristics e.g. hypercholesterolemia as dependent variables and NCCP or control as the predicting variable. Then multivariate regression was carried out with clinical characteristics as dependent variables to adjust for age, sex and “consultations for chest pain in recent years”. The level of significance was set at *p* < 0.05. At the evaluation to increase statistical power the matching was broken due to the non-responders.

## Results

Table 1 shows mortality rates almost 6 years after the initial investigation by the GPs. All-cause mortality and causes of death did not differ significantly between the groups. The groups did not differ with respect to demographic data (Table 2). Patients with NCCP consulted their GPs for chest pain more frequently (OR 4.45 95 % CI 3.24–6.10) (Table 3) and they more often experienced chest pain during the last 6 months (OR 3.34 95 % CI 2.41–4.62). The groups did not differ with respect to self-reported acute CHD and stable angina pectoris (Table 3). Patients with NCCP had more angina pectoris, but the difference disappeared when adjusting for, “consultations for chest pain in recent years”. Patients with NCCP more often stated that they had increased cholesterol levels but the difference was insignificant when adjusting for “consultations for chest pain in recent years” (OR 1.44 95 % CI 1.00–2.08). NCCP proved to be associated with hypertension (OR 1.86 95 % CI 1.32–2.60) (Table 3). In the multivariate analysis no gender difference was found with respect to hypertension (OR 0.98 95 % CI 0.73.1.36). It is evident that NCCP patients consumed more β-blockers (*p* < 0.001), thiazides (*p* = 0.004), aspirin (*p* = 0.013) and long-acting nitrates (*p* = 0.002). They further had more prescriptions for acid-related disorders (*p* = 0.014) and obstructive pulmonary disease (*p* < 0.001) (Table 4).

**Table 1:** All-cause mortality and causes of death for the patients with non-coronary chest pain (NCCP) and the population controls

	NCCP (*n* = 382) (%)	Controls (*n* = 746) (%)	*P* value
All-cause mortality	4.2	3.8	0.734
Cause of death			
Cerebrovascular diseases	0.3	0.5	0.668
Coronary heart disease	0.5	1.1	0.509
Other^a^	2.9	1.7	0.222
Other diseases of the circulatory system	0.5	0.4	1.000

^a^Accidents, neoplasms and diseases of the genitourinary, nervous and respiratory systems

**Table 2:** Demographic characteristics for patients with non-coronary chest pain (NCCP) and population controls, according to the postal survey distributed in 2005

	Patients with NCCP (*n* = 279) (%)	Population controls (*n* = 530) (%)	*p*-value
Age at inclusion			
35–49 years (%)	27	29	0.778
50–59 years (%)	37	38	
60–69 years (%)	24	21	
70–79 years (%)	13	12	
Sex (M/F) (%)	43/57	43/57	0.842
Married (%)	79	75	0.160
Education			
Compulsory education^a^ (%)	49	41	0.054
Upper secondary school (%)	34	42	
University (%)	17	18	
Current smokers (%)	13	16	0.383
Current tobacco snuffers (%)	10	10	0.940

^a^Less than 10 years in school

**Table 3:** Self-reported clinical characteristics of patients with non-coronary chest pain (NCCP) and population controls after 6 years follow-up

	NCCP (*n* = 279) (%)	Controls (*n* = 530) (%)	*p*-value^a^	OR^a^ (95 % CI)	*p*-value^b^	Adjusted OR^b^ (95 % CI)
Consultations for chest pain in recent years^c^	65	30	<0.001	4.45 (3.24; 6.10)	N.A.	N.A.
Chest pain symptoms during the last 6 months	45	20	<0.001	3.34 (2.41; 4.62)	N.A.	N.A.
Self-reported angina pectoris^d^	8.6	4.7	0.01	2.44 (1.28; 4.62)	0.39	1.36 (0.68; 2.73)
Acute coronary syndromes^e^	3.6	2.6	0.45	1.37 (0.60; 3.13)	0.43	0.70 (0.28; 1.70)
Stable angina pectoris^e^	2.2	1.5	0.51	1.43 (0.49; 4.18)	0.67	0.78 (0.26; 2.38)
Diabetes mellitus^d^	7.7	8.4	0.73	0.91 (0.53; 1.56)	0.52	0.82 (0.45; 1.49)
Hypercholesterolemia^d^	30	20	0.001	1.76 (1.26; 2.47)	0.05	1.44 (1.00; 2.08)
Hypertension^d^	51	34	<0.001	2.04 (1.51; 2.78)	<0.001	1.86 (1.32; 2.60)

*N.A.* = Not Applicable

^a^Univariate logistic regression

^b^Multivariate logistic regression. Estimates adjusted for age, sex and “Consultations for chest pain in recent years”. Goodness-of-fit was measured with Nagelkerke’s R^2^

^c^No specific time limit was given for “recent years”

^d^Ever informed by a physician of having the disease

^e^Information is validated through medical records

**Table 4:** Medication taken by patients with non-coronary chest pain (NCCP) and population controls after 6 years follow-up according to the postal survey distributed in 2005

	NCCP (*n* = 279) (%)	Controls (*n* = 530) (%)	*p*-value
ACE inhibitors	8.6	5.7	0.111
A_II_ inhibitors	6.8	4.3	0.132
β-Blockers	27	16	<0.001
Ca^2+^-Blockers	8.6	6.6	0.299
Furosemide	6.5	6.0	0.816
Thiazides	15	8.5	0.004
Aspirin	18	11	0.013
Long-acting nitrates	4.7	1.1	0.002
Statins	13	9.2	0.078
Agents for acid-related disorders^a^	13	2.8	<0.001
Analgesics and NSAID	7.9	5.5	0.179
Anti-diabetic medication	4.7	4.2	0.735
COPD agents	7.5	3.6	0.014
Anti-depressants	7.9	6.0	0.317
Hypnotics and sedatives	10	8.1	0.358

*ACE* angiotensin-converting enzyme, *A*_*II*_ angiotensin II, *NSAID* non-steroidal anti-inflammatory drugs, *COPD* chronic obstructive pulmonary disease

^a^Antacids, H_2_-receptor antagonists and proton pump inhibitors

## Discussion

The findings of this long-term follow-up of almost 6 years of NCCP patients in primary care suggest that these patients do not develop CHD more frequently than a population control group matched for age, gender and residential area (Table 3). The results also suggest that NCCP does not affect mortality (Table 1). It is further apparent that the condition often lasts for many years and associates with hypertension (Table 3).

In this study the NCCP group was selected prospectively and the controls retrospectively. In 2005, at study end the groups did not differ with respect to the clinical characteristics given in Table 2. They could be different at inclusion and more importantly the groups may diverge regarding clinical features not being investigated by us. At inclusion the index group was painstakingly investigated by the GPs to exclude CHD whereas the controls did not pass such an investigation. The handling differs between groups making it tenable that some controls had subclinical CHD unknown to us. The bias most likely affects mortality and CHD frequency among controls.

The most appropriate approach is to omit unsuitable participants before inclusion and to use similar exclusion strategies for both groups. It is further hazardous to leave out participants post-hoc after groupings have been defined. Limited resources made it impossible for the GPs to investigate 784 apparently healthy controls with respect to subclinical CHD. As a compromise, in this study participants having pre-existing CHD were identified and excluded in 2005. Individuals with severe conditions more easily recall details about their disease and clinical data shown in Table 3 are most likely compromised by recall biases. It is also tenable that individuals frequently seeking medical attention have better knowledge about risk factors for CHD.

We validated medical records if subjects noted CHD in the postal questionnaire and excluded participants if hospital charts verified such a condition prior to inclusion. Especially among non-responding controls such cases may be unidentified. Postal questionnaires with a high degree of certainty exclude previous myocardial infarction [15, 16] but it is reasonable that they are less accurate in identifying angina pectoris. However, self-reported angina pectoris matches data obtained from medical records reasonably well [17]. Consequently, the review of hospital charts was limited to subjects who stated that they had a diagnosed CHD. To include symptoms of current relevance the survey asked for chest pain occurring during the last 6 months. It is desirable to match the groups for clinical data such as hypertension as well. The Swedish National Population Registry does not contain such information making the undertaking impossible.

The NCCP condition associates with increased all cause long-term mortality [5, 6]. NCCP patients with a normal exercise test had lower mortality due to CHD after 6 years than a general population control group [18]. We failed to verify both findings (Table 1). Possible explanations include that the GPs had easy access to exercise testing and myocardial perfusion scintigraphy. A previous study showed that patients with NCCP in 56 % of cases had persistent symptoms after 6 months [4]. In our study, NCCP-patients reported chest pain symptoms after as long as 6 years in 45 % of cases with a more than three-fold increased risk as compared with population controls (Table 3). The current work also reveals that hypertension is more widespread among patients with NCCP (Table 3) but contrary to a previous study we failed to show gender differences with respect to hypertension [13]. Patient newly diagnosed with NCCP frequently use drugs for acid-related disorders [5]. It is in line with our findings. Chest wall syndromes are common in primary care [19] but in our hands analgesic consumption was low in both groups (Table 4). NCCP patients with repeated healthcare consultations have a high incidence of depressive symptoms and cardiac anxiety [12]. It disagrees with current findings as anti-depressants or sedatives prescriptions did not differ between groups (Table 4). The persistence of complaints and increased consultation rates suggest that NCCP belongs to the group of medically unexplained physical symptoms. A linkage between NCCP, health care utilisation and alexithymia, i.e. a difficulty identifying or verbalizing emotions has been demonstrated among men. Alexithymia was also shown to be increasingly stable over time. Likewise, there was a linkage between NCCP, health care utilisation and anxiety sensitivity among women [20]. These circumstances underline the importance of a positive diagnosis of NCCP at an early stage, to prevent unnecessary investigations and costly health care utilization [9].

Initially, the study groups were matched for age, gender and residential area. Due to the non-responders to the postal questionnaire many NCCP patients were left without control counterparts. To increase statistical power the matching was then broken. Death certificates give the final cause of death in conjunction with underlying conditions (*n* = 2). They also document other medical conditions contributing to death. However, the quality of death certificates vary but it is unlikely that the bias differ between study groups. In this case the primary care centers catchment areas are identical to the parishes used by the Swedish National Population Registry making it possible to select patients and controls from the same geographical area. Participants from 1 suburban and 2 rural healthcare centres were included. It is possible that GPs supporting a population with many social problems show less favourable results.

Angina pectoris may have a different meaning to the population than the strict medical sense and patients’ perceptions depend on the number of chest pain consultations. Further, the belief of having augmented cholesterol levels and hypertension also increases by more medical consultations. Cardiovascular medication is frequently administered when CHD is suspected. GPs may later continue the therapy, for example with β-blockers, due to increased blood pressure. It is also possible that GPs do not delete prescriptions of long-acting nitrates when the clinical evaluation fails to confirm the suspicion of CHD.

## Conclusions

This study suggests that patients with NCCP do not have an enhanced risk for developing CHD but they demonstrate increased prevalence of hypertension. Individuals with NCCP have more prescriptions for β-blockers, thiazides, aspirin and long-acting nitrates. They also had more drugs for acid-related disorders and obstructive pulmonary disease. The condition often continues for many years and leads to repeated medical consultations.
